# Antigen-specific response of CD4^+^ T cells and hepatic lymph node cells to *Fasciola hepatica*-derived molecules at the early and late stage of the infection in sheep

**DOI:** 10.1186/s13567-021-00963-5

**Published:** 2021-07-02

**Authors:** Raúl Pérez-Caballero, F. Javier Martínez-Moreno, Yolanda Corripio-Miyar, Tom N. McNeilly, Krystyna Cwiklinski, John P. Dalton, Rafael Zafra, José Pérez, Álvaro Martínez-Moreno, Leandro Buffoni

**Affiliations:** 1 Department of Animal Health (Parasitology and Parasitic Diseases), Faculty of Veterinary Medicine, University of Córdoba, Campus de Rabanales, Ctra.Madrid-Cádiz, km 396, 14014 Córdoba, Spain; 2 Moredun Research Institute, Pentlands Science Park, Bush Loan, Penicuik, EH26 0PZ Midlothian UK; 3 Molecular Parasitology Laboratory, Centre for One Health and Ryan Institute, National University of Ireland Galway, Galway, Ireland; 4 Department of Anatomy, Comparative Pathology and Toxicology, Faculty of Veterinary Medicine, University of Córdoba, Campus de Rabanales, Ctra. Madrid-Cádiz, km 396, 14014 Córdoba, Spain

**Keywords:** Hepatic lymph nodes, CD4^+^ T cells, cytokines, *F. hepatica*, sheep, ruminants

## Abstract

The immunomodulatory capacity of *F. hepatica* antigens is probably one of the main reasons for the development of a driven non-protective Th2 immune response. In this study, we analysed the cellular response of hepatic lymph node cells and CD4^+^ T cells in terms of proliferative response, efficiency of antigen presentation and cytokine production, to *F. hepatica*-derived molecules, at early and late stages of the infection. Thirty-one sheep were allocated into five groups and were slaughtered at 16 dpi and 23 wpi. In order to analyse antigen-specific response, the following *F. hepatica* recombinant molecules were used: rFhCL1, rFhCL2, rFhCL3, rFhCB1, rFhCB2, rFhCB3, rFhStf-1, rFhStf-2, rFhStf-3 and rFhKT1. A cell proliferation assay using hepatic lymph node cells and an antigen presentation cell assay using CD4^+^ T cells were performed. At 16 dpi, all molecules but rFhStf-2 and rFhKT1 elicited a significant cell proliferative response on hepatic lymph node cells of infected animals. At both early and late stage of the infection, antigen presentation of rFhCB3 and rFhCL2 resulted in higher stimulation index of CD4^+^ T cells which was IL-2 mediated, although no statistically significant when compared to uninfected animals. Significant cytokine production (IL-4, IL-10 and IFN-γ) was conditioned by the antigen-specific cell stimulation. No CD4^+^ T cell exhaustion was detected in infected sheep at the chronic stage of the infection. This study addressed antigen-specific response to *F. hepatica*-derived molecules that are involved in key aspects of the parasite survival within the host.

**Supplementary Information:**

The online version contains supplementary material available at 10.1186/s13567-021-00963-5.

## Introduction

Fasciolosis by *Fasciola hepatica* is a major concern as it causes a serious economic impact on livestock and public health, mainly in endemic regions of Latin America, Asia and Africa [1]. Animals affected by the liver fluke suffer from a severe hepatitis, which may lead to significant meat, milk and wool production loss.

The parasite releases a variety of molecules with immunomodulatory capacity on the immune response of the host [2–7]. The effects of these molecules on the host may differ depending on many aspects such as the presence of immunodominant epitopes, the type of immune cell subset they interact with, and/or the outcome during antigen presentation by antigen-presenting cells (APC). In addition, the protein profile of *F. hepatica* molecules that are released within the secretome diverges from immature-juvenile stages to adult stage, as does the immune responses throughout the course of infection [8–10].

The immunomodulatory effects of *F. hepatica* molecules on its host have been extensively investigated. The immune response elicited by the parasite develops towards a non-protective profile even from the initial stages of the infection, characterised by a Th2 phenotype which progresses into a regulatory phenotype as the infection becomes chronic [11–13]. Although there is still a lack of knowledge about the antigen-specificity of the T cell response, previous studies in mice and ruminants suggested that *F. hepatica* induces Treg cells with TGF-β and IL-10 mediated immunosuppressive activity [14–16]. Understanding the antigen-specificity and reactivity of T cells is key to fully comprehend immune response during fasciolosis.

One site where adaptive immune cell activation occurs during *F. hepatica* infection is the hepatic lymph node (HLN) [17, 18]. Once migrating juvenile flukes have entered the liver, activation and expansion of B and T lymphocytes within the HLN occurs with upregulation of interleukin (IL)-4 gene expression, which is in agreement with the development of a local T-helper type 2 response [18, 19]. More recently, it has been shown that increased numbers of activated CD4^+^ T cells are present within the HLN of cattle at 13 weeks post-infection, but these cells are poorly responsive to re-stimulation with *F. hepatica* antigen suggesting T cell exhaustion [20]. Thus, a complex pattern of antigen-specific activation of HLN CD4^+^ T cells in early infection may progress to T cell exhaustion in chronic infection.

Studies of adaptive immunity in liver fluke infection have been largely restricted to using complex *F. hepatica* antigen preparations such as total adult antigen [20–23] and, therefore, specific antigen-specificities of the CD4^+^ T cell response are largely unknown. Consequently, the aim of this study was to analyse cellular response of HLN cells and CD4^+^ T cells to specific *F. hepatica* secreted antigens derived from the migratory stages of *F. hepatica*. Studies focused on early and late stages of infection in sheep in order to investigate potential T cell exhaustion at later stages of infection.

## Materials and methods

### Animals and experimental design

Thirty-one 6-month-old male Merino-breed sheep obtained from a liver fluke-free farm were used for both experimental infection trials. Prior to the beginning of the experiment, animals were treated with Ivermectin (Noromectin®, Karizoo, Barcelona, Spain) and Diclazuril (Rumicox®, Esteve, Barcelona, Spain) and subsequently tested negative for parasite eggs and *Eimeria* spp. oocysts by faecal examination. Sheep were confirmed free from liver fluke infection by testing for parasite eggs in faeces three times at 4-day intervals using a zinc-sulphate-based flotation technique, and serologically using a *F. hepatica* specific ELISA [24]. Animals were housed in covered pens and fed daily with hay and commercial pelleted ration.

Animals were randomly allocated into five groups as follows: for trial 1, two groups of eight animals each were used: G1 was uninfected (negative control) and animals from G2 were infected as detailed below and slaughtered at 16 days post-infection (dpi). Trial 2 involved three experimental groups, each containing five animals: G3 was uninfected, G4 and G5 were infected as detailed below and slaughtered at 16 dpi and 23 weeks post-infection (wpi), respectively.

Animals from G2, G4 and G5 were orally challenged with a dose of 150 metacercariae of *F. hepatica* (Ridgeway Research Ltd., St Briavels, UK) within gelatine capsules, using a dosing gun. Sheep were euthanised by an intravenous injection of T61® (Intervet, Barcelona, Spain).

Two experimental trials were performed as follows: the first trial consisted of uninfected (G1) and infected animals (G2) that were slaughtered at 16 dpi, and was aimed at analysing the cellular response of lymphocytes from HLN to stimulation by an array of *F. hepatica* molecules (rFhCL1, rFhCL2, rFhCL3, rFhCB1, rFhCB2, rFhCB3, rFhStf-1, rFhStf-2, rFhStf-3, rFhKT1) which are known to be expressed by either newly excysted juveniles (NEJ), immature or mature stages of the parasite [25–30] (Additional file 1), and to determine the CD4^+^ T cell population during the early stage of the infection. For the second trial, two *F. hepatica* molecules (rFhCL2, rFhCB3) which showed a significant increase of the stimulation index in response to antigen stimulation in trial 1, were investigated further in an antigen presentation cell study during the early and late stages of the infection (16 dpi and 23 wpi, respectively). The experiment schedule is summarised in Additional file 2.

These experiments were performed in accordance with the Spanish and the European Union regulations (L32/2007, RD53/2013, Directive 2010/63/EU), and in accordance with the University of Córdoba Bioethics Committee (code no. 1118).

### Recombinant protein expression of *F. hepatica* antigens

Recombinant protein production was carried out for ten *F. hepatica* antigens that display differential expression across the life cycle; namely, three *F. hepatica* cathepsin L peptidases (rFhCL1, rFhCL2, rFhCL3; [31]), three cathepsin B peptidases (rFhCB1, rFhCB2, rFhCB3; [9, 32]), three stefin cysteine peptidase inhibitors (rFhStf-1, rFhStf-2, rFhStf-3; Cwiklinski, Drysdale & Dalton, unpublished) and Kunitz type inhibitor (rFhKT1, [29]). Recombinant expression was carried out in the methylotrophic yeast *Pichia pastoris* with a C-terminal His-tag and proteins were purified using a previously described protocol [31]. Protein concentration and purity were verified by Bradford Protein Assay (Bio-Rad) and by 4–20% SDS-PAGE gels (Bio-Rad) stained with Biosafe Coomassie (Bio-Rad), respectively.

### Isolation of hepatic lymph node cells

A 2-cm section of HLN was obtained during necropsy and added to sterile containers with 20 mL of HBSS (Gibco™) containing 1% of antibiotic–antimycotic solution (Sigma-Aldrich®, Spain). Under sterile conditions, connective tissue was removed, and lymph node samples were minced in Hank’s Balanced Salt Solution (HBSS) until complete maceration of tissue was obtained. The resulting suspension was transferred with a Pasteur pipette through a 70 µm sterile cell-culture strainer (Fisherbrand®) into a sterile 50 mL tube. The suspension was centrifuged at 250 *g* for 5 min at room temperature (RT), erythrocytes were lysed by adding 5 mL of RBC lysis buffer (0.15 M NH_4_Cl, 10 mM KHCO_3_, 0.1 mM disodium EDTA, dH_2_O) for 3 min. Then, culture media (RPMI-1640 medium supplemented with 200mML-glutamine, 10 000 U/mL penicillin, 10 mg/mL streptomycin, Sigma-Aldrich®, Spain) was added to fill the 50 mL tube. After centrifugation, the supernatant was removed, the cells were enumerated and final cell concentration was adjusted to 3 × 10^7^ cells/mL. Cells were pelleted by centrifugation, re-suspended in freezing media (FCS + 10%DMSO) before being transferred into cryovials, placed into CoolCell™ (Sigma-Aldrich®, Spain) at −80 °C overnight and stored in liquid nitrogen until use.

### Cell proliferation assay of hepatic lymph node cells of trial 1

Hepatic lymph node cells were defrosted in a water bath at 37 °C until almost completely thawed. 1 mL of warm complete tissue culture medium (RPMI-1640 medium supplemented with 10% heat-inactivated foetal calf serum (HiFCS), 50 µM 2-mercaptoethanol, 2 mM L-glutamine, 100 U/mL penicillin, 100 µg/mL streptomycin and 5 µg/mL of gentamycin; Sigma-Aldrich®, UK) was gently added to each sample and then transferred to a 50 mL tube containing 3 mL of warm medium. Cells were washed twice with 10 mL of complete medium by centrifugation at 300 *g* for 10 min to eliminate cell debris. The cell pellet was re-suspended in 1 mL of complete medium, viable cells were counted using a hemocytometer, and each sample was then adjusted to 2 × 10^6^ cells/mL.

Lymphocyte stimulation assay (LSA) was carried out as follows. Briefly, 2 × 10^5^ HLN cells per well were incubated in Costar® flatbottom 96-well plates (Corning® Life Sciences, UK) in triplicate in a total volume of 200 μL containing 5 µg/mL of each *F. hepatica* recombinant antigen (see above), 5 µg/mL of ConA (positive control) and media only (negative control). Plates were incubated at 37 °C with 5% CO_2_ for 4 days and then, 50 µL of media from each well were collected and stored for cytokine analysis and replenished with 50 µL of media containing methyl-3H thymidine (0.5 μCi per well; Amersham Biosciences UK Ltd, Chalfont St. Giles, Buckinghamshire). The level of cell proliferation was measured by the incorporation of [^3^H] thymidine into DNA during the final 18 h of culture. Data are presented as the corrected counts per minute (ccpm) averaged over 3 min. Stimulation index (SI) was calculated by dividing the mean of the triplicate counts per minute by the mean of the relevant *F. hepatica* recombinant stimulated samples.

### Flow cytometry of CD4^+^ T cells from hepatic lymph node

In order to determine the percentage of CD4^+^ T cells, single colour flow cytometric analysis was performed using HLN cells from all animals. Briefly, following resuscitation, cells were re-suspended into FACS Buffer (PBS, 5% HiFCS and 0.02% sodium azide) and concentration adjusted to 10^7^ cells/mL and 100 µL per test aliquoted in a 96-well round-bottom plate. Cells were then blocked with 20% heat-inactivated Normal Goat serum for 15 min. Following centrifugation, supernatant was discarded and cells were incubated in dark at RT for 20 min with a predetermined concentration of anti-ovine CD4^+^ antibody (clone 44.38, BioRad) conjugated to Alexa Fluor 647 or 50 µL of FACS buffer was added to control samples (no antibody control). Cells were then washed twice by adding 100 µL of FACS buffer to all wells and centrifuged at 1500 rpm for 1 min and re-suspended into 100 µL of FACS buffer. Immediately prior to acquisition, 100 µL of 2 × dead cell stain Sytox Blue (Invitrogen, Life Technologies, USA) were added to all wells and a minimum of 20 000 events was acquired using a MACSQuant® Analyzer 10 (Miltenyi Biotech, Germany). Data analysis was carried out using the analysis software FlowJo®v10. The gating strategy consisted of an initial dead cell and doublet cell discrimination, followed by a live, single lymphocytes gate based on forward scatter (FSC-H) and side scatter (SSC-H). Finally, a CD4 gate was created using the no antibody control samples.

### Isolation of peripheral blood mononuclear cells (PBMC)

PBMC were used in antigen presentation cell assay (APCA) (see below) to assess CD4^+^ T cells response to APC. Before slaughtering, blood samples from animals of G3, G4 and G5 were taken for PBMC isolation. Twenty mL of blood were collected using vacutainer tubes containing lithium heparin (BD Vacutainer® LH 68 I.U.) by jugular puncture. Samples were centrifuged at 3000 rpm for 30 min with no brake in 30 mL tubes. After centrifugation, buffy coat was collected and diluted in 1:1 ratio with phosphate-buffered saline (PBS, Dulbecco A, Oxoid®). Cells and PBS mixture was gently overlaid onto an equal volume of Histopaque®-1077 (Sigma-Aldrich®) into 15 mL tubes and were centrifuged at 3000 rpm for 30 min with no brake at 4 °C. Buffy coat was then collected with a sterile Pasteur pipette and washed three times with PBS-EDTA (PBS + 6 mM EDTA) by centrifuging at 1500 rpm for 10 min. Cells were re-suspended in freezing media (RPMI 5%FCS + 10%DMSO) and transferred to cryovials. Samples were placed in CoolCell™ (Sigma-Aldrich®, Spain) at -80 °C overnight and preserved in liquid nitrogen until used.

### Antigen presentation cell assay of trial 2

The aim of this assay was to determine how efficiently CD4^+^ T cells from HLN of infected animals were able to respond to APC. To do this, CD4^+^ T cells from HLN were sorted and then incubated with irradiated PBMC obtained from the same animal (autologous PBMC), alongside with rFhCB3 or rFhCL2. Consequently, the efficiency of the antigen presentation was measured by the proliferation of the CD4^+^ T cells.

To sort the CD4^+^ T cells from HLN preparations from the second trial, cells from G3, G4 and G5 were stained as detailed above. Cell pellets were then re-suspended in 500 µL of media without HiFCS and CD4 cells were sorted in a FACSAria™ III at the Roslin Institute Imaging Facility (Edinburgh, UK). Once sorted, cells were washed and re-suspended in complete media at a concentration of 2 × 10^6^ cells/mL.

Autologous PBMC were resuscitated as detailed above and re-suspended at a concentration of 5 × 10^6^ cells/mL in complete media and irradiated at 60 Gy.

The APCA was performed by incubating 50 000 CD4^+^ T cells (responder cells) with 50 000 irradiated autologous PBMC (stimulator cells) alongside with 5 µg/mL of rFhCB3 or rFhCL2, 5 µg/mL of ConA (positive control) or media only (negative control) with and without 200 IU/mL of rhuIL-2. Reactions were set up in triplicate in U-well microtitre 96 well-plates in a total volume of 200 μL. After 4-day incubation at 37 °C with 5% CO_2_, 50 µL of media from each well were collected and stored for cytokine analysis and replenished with fresh complete medium containing methyl-3H thymidine (0.5 μCi per well) and after 18 h incubation, proliferation was determined as detailed above.

### Detection of cytokine production

Detection of cytokines (IL-4, IL-10 and IFN-γ) in supernatant from LSA of HLN cells from trial 1 (G1 and G2) and cytokines (IL-4 and IFN-γ) from the APCA from trial 2 (G3, G4 and G5) was performed by a capture-ELISA as follows: high-binding capacity ELISA plates (Immunolon 2HB, 96-well microtiter plates, ThermoFisher) were coated with 4 µL/mL of IL-10 antibody (CC318, BioRad) in 0.5 M Carbonate coating buffer or with 2 µg/mL of IFN-γ (MT17.1, #3119-1H, Mabtech) and IL-4 (bIL4-I, #3118-1H, Mabtech) capture antibodies in PBS, overnight at 4 °C. After five washes with washing buffer (PBS containing 0.05% Tween 20), plates were blocked with 100 µL/well for IL-4 and IFN-γ or 200 µL/well for IL-10 of blocking buffer (PBS + 0.05% Tween 20 + 3% BSA for IL-10 or PBS + 0.05% Tween 20 + 0.1% BSA for IL-4 and IFN-γ) and incubated at RT for 1 h. Wells were washed five times and 50 µL of each sample or standard diluted in dilution buffer were added and incubated at RT for 1 h for IL-10 or 2 h for IL-4 and IFN-γ. After five washes, 50 µL/well of biotinylated secondary antibody (1 µg/mL for IL-10; CC320b- BioRad; 0.5 µg/mL for IL-4,mAb bIL4-II, Mabtech and 0.25 µg/mL for IFN-γ, mAb MT307, Mabtech) were added and incubated for 1 h. After washing, 50 µL/well of streptavidin-HRP diluted 1:1000 in dilution buffer were added and incubated for 1 h at RT. Wells were washed and 50 µL/well for IL-4 and IFN-γ or 100 µL for IL-10 of SureBlue TMB peroxidase substrate (Insight Biotechnology, London, UK) were added to each well and incubated at RT for 10 min. The reaction was stopped by adding 50 µL/well of TMB stop solution (Insight Biotechnology, London, UK) and optical density was measured at 450 nm in a spectrophotometer (Magellan™, Tecan Multimode Readers). Standard curves were included in all plates and were constructed using seven serial dilutions of recombinant cytokines ranging from 6.25 to 400 pg/mL for IFN-γ (Mabtech AB); 31.25 to 2000 pg/mL for IL-4 (MabtechAB) and 0.206 to 13.2 BU/mL for IL-10 [33]. All values were blanked-corrected and concentrations were determined from standard curve.

### Statistical analysis

Statistical analysis was performed with GraphPad Prism v.6.0 software (GraphPad Software Inc., San Diego, CA, USA). Differences in the percentage, mean fluorescence intensity and total number of CD4^+^ T cells pre- and post-proliferation, the response to the different antigens between groups and the cytokine and antibody productions were determined by applying two-tailed Mann–Whitney U-test for non-parametric distributions. *P* values of 0.05 or lower were considered statistically significant.

## Results

### Experimental infection

To confirm that animals were successfully infected, all livers were carefully analysed during necropsy for the presence of hepatic lesions and parasites. In animals from the early stage infection (16 dpi, G2 and G4) gross hepatic lesions consisted of haemorrhagic-necrotic migratory tracts, whereas whitish-fibrotic tortuous tracts and thickening and enlargement of bile ducts were found in animals from the late stage of the infection (G5). Mature flukes from G5 were enumerated to obtain individual fluke burden (Additional file 3).

### CD4^+^ T cell population from HLN

**Figure 1:**
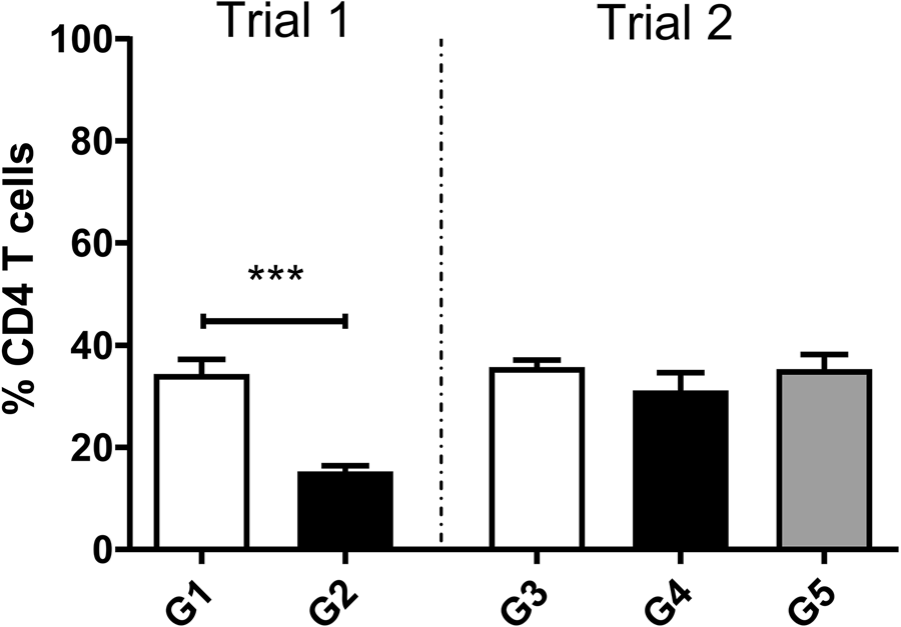
**CD4**^**+**^** T cell population from HLN of uninfected and infected sheep.** Percentage of CD4^+^ T cell from HLN in uninfected and infected animals after 20 000 events, by flow cytometry. Each column represents mean values, bars show standard error. Mann–Whitney U-test was used to compare data from infected animals to uninfected animals. Statistical differences are represented by asterisks (***P = 0.0002).

The percentage of CD4^+^ T cells in the HLN from the early stage (trial 1 and trial 2) and late stage of the infection (trial 2) were analysed by flow cytometry. Following gating for single and live cells, the results were expressed as the percentage of lymphocytes which were CD4^+^ in each sample and are shown in Figure 1.

In trial 1, the percentage of CD4^+^ T cell in uninfected and infected animals was 34.40% and 15.30%, respectively. In sheep from trial 2, CD4^+^ T cells represented 35.74%, 31.18% and 35.34% of cells in uninfected, early-stage infected and late-stage of infection, respectively. The comparative analysis showed significantly fewer CD4^+^ T cells in G2 when compared to G1 (*P* = 0.0002), while no significant differences were observed between groups of trial 2.

### Effect of *F. hepatica*-derived antigens on proliferation of HLN cells

**Figure 2:**
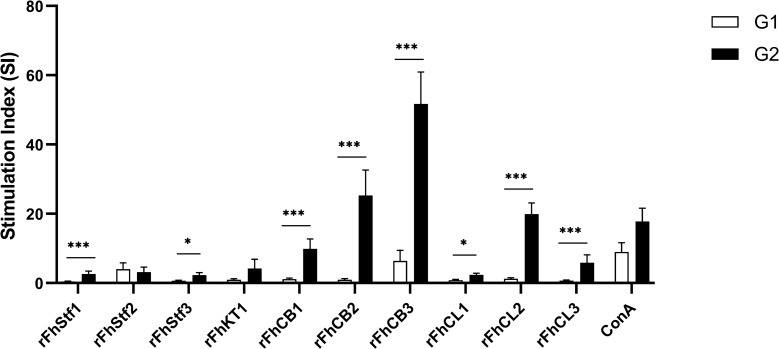
**Proliferative response of hepatic lymph node cells from infected and naïve animals.** HLN cells of *F. hepatica* infected animals during the early stage of the infection were treated with different *F. hepatica*-derived antigens for a 4-day period, and proliferative response was determined by [3H] thymidine incorporation into DNA during an 18-h period. Each column represents mean values of stimulation index (shown below columns), bars at each point show standard error. Statistical differences (Mann–Whitney U-test) are represented by asterisks (*P < 0.05, ***P < 0.001).

Results of the proliferation induced by *F. hepatica* antigens on HLN cells from trial 1 are shown in Figure 2. As measured by [^3^H] thymidine incorporation into DNA, all antigenic treatments except rFhStf2 elicited cell proliferation at 16 dpi when compared to the control group. rFhCB3 elicited the highest SI. The comparative analysis showed statistical differences between uninfected and infected animals (G1 and G2, respectively) in eight out of ten antigens: *P* = 0.0006 for rFhStf1, rFhCB1 and rFhCB3; *P* = 0.0002 for rFhCB2, rFhCL2 and rFhCL3; and *P* = 0.0148 for rFhStf3 and rFhCL1.

When compared to controls, the data for each treatment showed that all *F. hepatica* molecules except rFhStf2 elicited a 26.46- to 2.91-fold increase in cell proliferation ratio in infected animals. In brief, cell proliferation was elevated by 26.46, 15.51, 8.69, 8.61, 8.11, 5.41, 4.34, 3.47 and 2.91 fold for rFhCB2, rFhCL2, rFhCB1, rFhCL3, rFhCB3, rFhStf1, rFhKT1, rFhStf3 and rFhCL1, respectively. By contrast, no increase in cell proliferation index was observed for treatment with rFhStf2 in infected sheep.

### Effect of antigen presentation of rFhCB3 and rFhCL2 on CD4^+^ T cells from HLN

**Figure 3:**
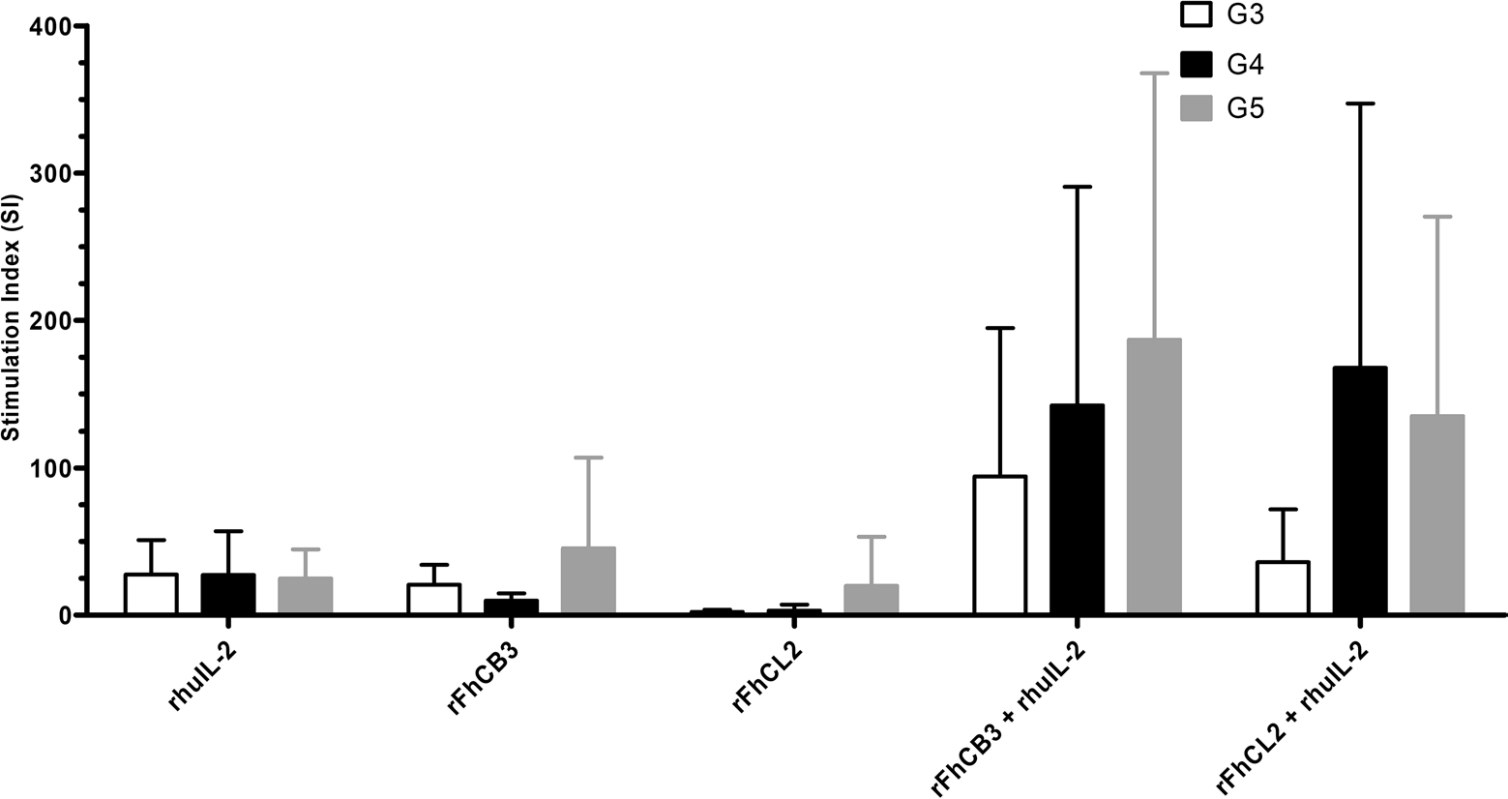
**CD4**^**+**^** T cell response during the antigen presentation cell assay.** Columns represent mean values per group (± SD) of stimulation index during trial 2. rFhCB3 and rFhCL2 were used for antigen presentation by autologous irradiated PBMC. Bars show standard error. No significant differences were detected between groups, whatever the treatment.

An APCA which included CD4^+^ T cells (responders) and irradiated PBMC (stimulators) from uninfected and infected animals during the early and late stage of the infection (G3, G4 and G5) was performed to assess efficiency of antigen presentation to CD4^+^ T cell following stimulation with rFhCB3 and rFhCL2. The results, measured by the proliferation of the CD4^+^ T cells, are presented in Figure 3 and show that antigenic stimulation elicited a different pattern between groups in terms of cell proliferative response. Animals during the early and late stage of the infection (G4 and G5, respectively) exhibited higher levels of SI than uninfected animals for both antigenic stimulations with rFhCB3 and rFhCL2 when rhuIL-2 was added, though no significant differences were detected between groups. At the early stage of the infection, cells showed a lower response to rFhCB3 than to rFhCL2, which contrasted with that observed at the late stage of the infection. When results from the control group were analysed (G3), a higher non-significant SI was observed for rFhCB3.

When antigen presentation was performed without rhuIL-2, the overall response was scarce and limited. Animals from the late stage of the infection displayed the most elevated response, yet no major variations were observed between groups.

### Cytokine response of HLN cells after stimulation with *F. hepatica*-derived molecules

**Figure 4:**
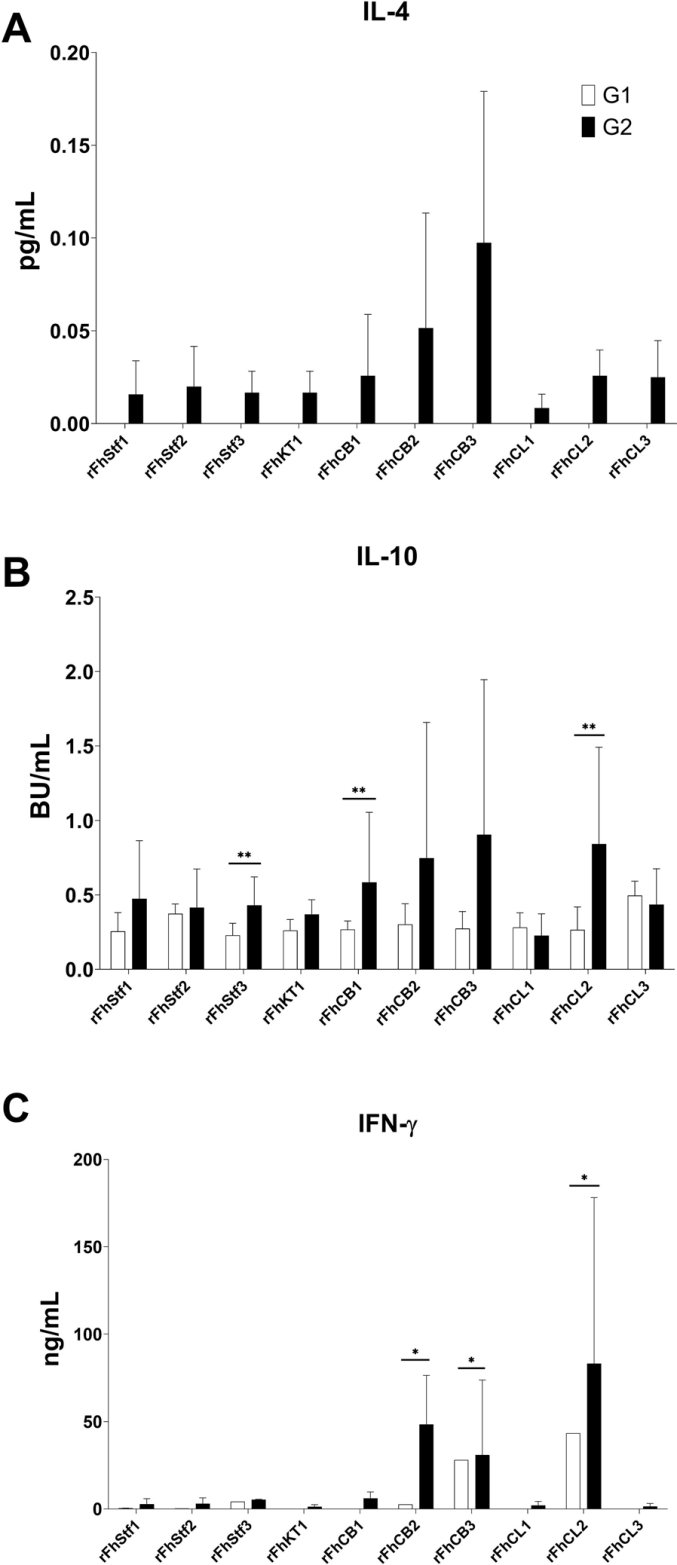
**Cytokine responses of hepatic lymph nodes of naïve and early-stage infected animals.** Cytokine production analysed from supernatants during LSA with HLN cells stimulated with *F. hepatica* molecules at the early stage of the infection (16 dpi) in uninfected (G1, white columns) and infected (G2, black columns) sheep. Each column represents the mean values per group (± SD), bars indicate standard error, asterisks indicate significant differences (*P < 0.05, **P < 0.01, ***P < 0.001) between groups. No IL-4 production was detected as results were below the detectable limits of ELISA.

Cytokine production (IL-10, IL-4 and IFN-γ) by HLN cells was analysed from supernatants originated from LSA in samples from early *F. hepatica* infection (G1 and G2) following stimulation with *F. hepatica*-derived molecules. Results are expressed as mean per group and are shown in Figure 4.

With respect to IL-4 (Figure 4A), no production was detected in both uninfected and infected groups. Production of IL-10 (Figure 4B) showed statistically significant differences for rFhStf3 (*P* = 0.007), rFhCB1 and rFhCL2 (*P* = 0.0019). As for IFN-γ, production was significantly elevated for rFhCB2 (*P* = 0.0298), rFhCB3 (*P* = 0.0135) and for rFhCL2 (*P* = 0.024) only in infected animals (Figure 4C); the highest production was detected for rFhCL2.

### Cytokine response during APCA after stimulation with rFhCB3 and rFhCL2

**Figure 5:**
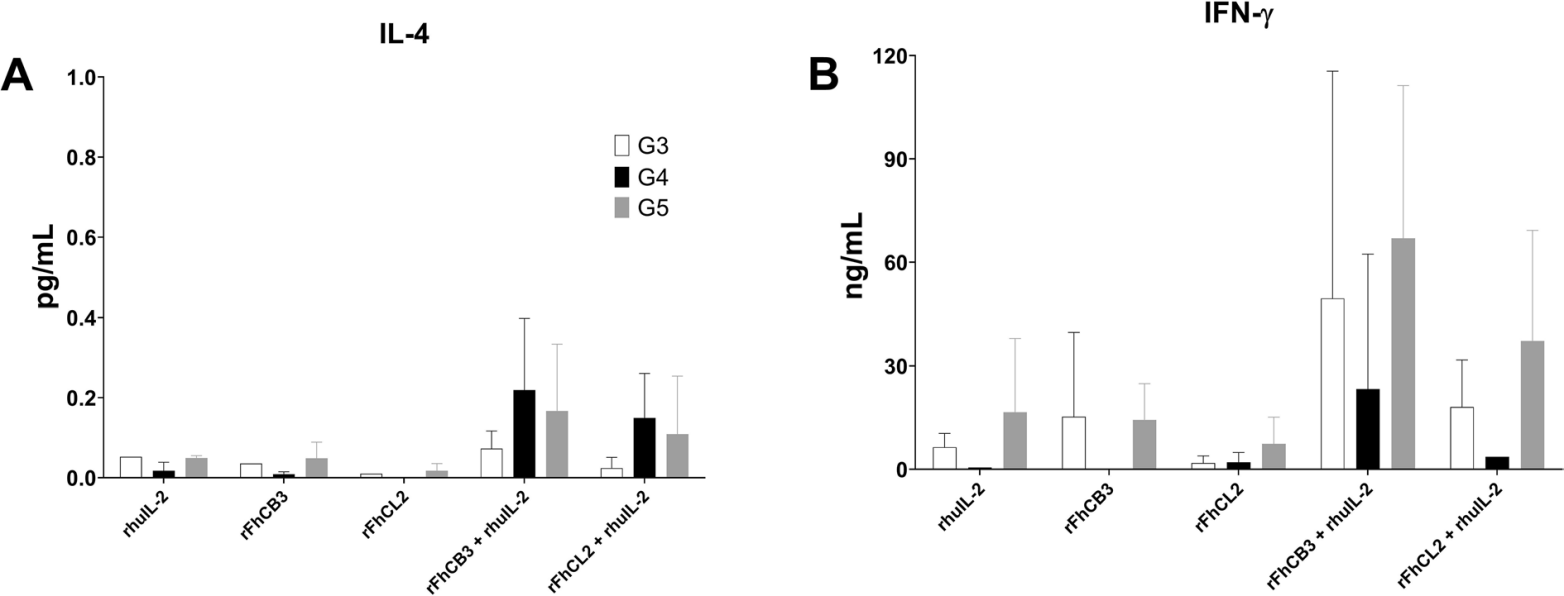
**Production of IL-4 and IFN-γ from CD4**^**+**^ **T cells after antigen presentation cell assay.** Production of IL-4 (A) and IFN-γ (B) analysed from supernatants during APCA. CD4^+^ T cells were cultured with autologous irradiated PBMC and antigen presentation of rFhCB3 and rFhCL2 were analysed in uninfected (G3) and infected sheep at 16 dpi (G4) and 23 wpi (G5). Each column represents mean values per group (± SD), bars indicate standard error. No IL-4 production was detected as results were below the detectable limits of ELISA. No significant differences were detected between groups for IFN-γ production.

Production of IL-4 and IFN-γ was determined from supernatants obtained from APCA after antigen presentation with rFhCB3 and rFhCL2 (Figure 5).

Overall, no production of IL-4 was detected in any of the groups regardless of the antigen treatment (Figure 5A). With regard to IFN-γ production (Figure 5B), the highest level was observed when cells from the late stage of the infection were treated with either *F. hepatica* molecules alongside rhuIL-2. In animals from 16 dpi, a lower production was detected in comparison to uninfected control sheep. When rhuIL-2 was not present during the stimulation, IFN-γ production was insignificant in all groups. No statistically significant differences were detected between groups for both cytokine production when data were compared.

## Discussion

Understanding cellular mechanisms controlling the adaptive immune response during fasciolosis and leading to a biased-Th2-non effective response is still a major goal. In our study, we experimentally challenged sheep with *F. hepatica* to determine the dynamic of CD4^+^ T cell population from HLN during the early and late stages of the infection, and we selected molecules that are expressed either by immature or mature stages of the parasite to explore specific cell reactivity of HLN cells.

In the first trial, we observed a significant in vivo decrease of the CD4^+^ T cell population during the early stage of infection, which was not detected in the second trial. These striking differences observed between trials, together with the lack of influence on the abundance of CD4^+^ T cells in HLN during early and late stage, might be due to individual differences in the adaptive immune response and T cell recruitment. In previous studies in goats, we have reported a significant decrease in CD4^+^ T cells from PBMC during chronic stage [34] which differed from the observation in the peritoneal cavity in sheep [35] and rats [36], suggesting a host-dependent mechanism regulating T cell motility.

We also performed in vitro studies to analyse proliferative responses by HLN lymphocytes and CD4^+^ T cells in response to *F. hepatica*-derived molecules. First, we focused on characterising cell reactivity to a variety of molecules produced by both the juvenile-migrating and adult stages of *F. hepatica* at the early stage of the infection, and we found that HLN cells from infected sheep elicited increased proliferative responses for all molecules with the exception of rFhStf2. In addition, we observed no significant difference in the SI between infected and uninfected cells exposed to rFhStf2 and rFhCB3, probably due to an enhanced cell proliferation of the uninfected cells, which may suggest a non-specific effect caused by *F. hepatica* antigens. These results suggest a major influence of rFhCB2 and rFhCB3 on the immune response during the initial stage of the infection. It is known that both peptidases are associated with newly excysted juvenile (NEJ) secretome profile [9, 32], and therefore may temporarily behave as immunodominant molecules in terms of antigen-specific response. However, further studies are required to elucidate this hypothesis.

Among cathepsin L family, we observed a significant increase in cell proliferation in response to rFhCL2 during early infection. This is somehow expected since FhCL2 is associated with both the immature (21 days) and adult stage of the parasite [10, 32], and corresponds to our early immune response analysis at 16 dpi.

Kunitz-type molecule was firstly identified in *F. hepatica* by Bozas et al. [37] and was further characterised by Smith et al. [29]. It is associated with the gut and parenchymal tissues of the juvenile stage, and it is thought to play a role on parasite immunomodulatory capacity by decreasing dendritic cell activation [38] and inhibiting cysteine peptidases of antigen-processing immune cells [29, 30]. Interestingly, our study showed that cell response to rFhKT1 was limited, indicating a minor role of FhKT1 during the early host-parasite interaction. Similar observations might be attributed to the *F. hepatica* stefins. These proteins, which were initially reported as cystatins in newly excysted juveniles (NEJ) and adult stages of *F. hepatica* by Khaznadji et al. [39], are also cysteine peptidase inhibitors believed to play a role on regulation of proteinases involved in fluke digestion and reproduction [32, 40, Cwiklinski, Drysdale & Dalton, unpublished]. Although significant differences were detected between uninfected and infected animals for rFhStf1 and rFhStf3, the overall antigenic stimulation of HLN cells to *F. hepatica*-derived stefins did not elicit major cell proliferative activity, suggesting a lack of influence on the early local adaptive immune response.

Down-regulation of cell proliferative response to helminth molecules have been reported [41] and *F. hepatica* excretory-secretory products (ESP) were shown to reduce proliferation in sheep and rat lymphocytes [42, 43]. Overall, our results contrast with previous reports in which *F. hepatica-*derived antigens showed a suppressive or lack of antigen-specific cell proliferation of lymphoid cells [4, 20, 21, 43–45]. However, most of those studies were performed either with cells from naive animals or cells obtained during the chronic stage of the infection, when the immune response is already biased towards a non-effective Th2-profile [3, 11, 46]. Furthermore, they employed *F. hepatica* antigenic cocktails such as excretory-secretory products (FhESP) or liver fluke homogenate (LFH) where complex antigenic interaction might affect cell proliferative response in a different way. Indeed, in cattle, a strong proliferative response of HLN cells was reported when whole fluke antigen was used [47]. Additionally, Zhang et al. [48] using FhESP observed an increase in the proliferation index of PBMC at the early stage of the infection in sheep, which contrasted with the decreasing trend as infection became chronic.

When we analysed the response of CD4^+^ T cells to rFhCB3 and rFhCL2 in trial 2, we observed a lack of proliferation, an effect that was reversed with the addition of exogenous rhuIL-2 to cell cultures in both naive and immunised sheep, although not statistically significant. This result is in agreement with recent reports in which a reduced proliferative activity of CD4^+^ T cells was elicited by *F. hepatica* antigens, which was overturned by the addition of IL-2 [20, 23]. Considering the lack of response in antigen-only cells and the increase of the SI observed in antigen plus rhuIL-2 cells, we suggest this enhanced cell response might be attributable to rhuIL-2, which highlights the key role IL-2 plays on T cell responsiveness, as previously reported [49].

Nevertheless, the rhuIL-2-induced CD4^+^ T cell response was higher in infected groups than in uninfected animals, indicating a higher cell capacity to proliferate in response to rFhCB3 and rFhCL2. The lack of statistically significant differences between infected groups and antigenic treatments suggest the time course of the infection might not be a relevant factor for rFhCB3 and rFhCL2 as antigen-specific cell stimulants on local adaptive immune response. However, we observed high individual variations within groups, which could also explain the absence of significant differences.

The analysis of cytokine production by HLN cells from infected sheep showed that rFhCB3, rFhCB2 and rFhCL2 induced the highest level of IL-10 and IFN-γ secretion, whereas a lack of production was observed for IL-4, which suggests these peptidases might be able to drive Th1 (IFN-γ) and Th2 (IL-4, IL-10) responses during the early stage of the infection. A similar observation was recently reported for *F. gigantica* cathepsin B protein which was able to enhance production of IL-10 and IFN-γ by goat PBMC [50]. In HLN cells from *F. hepatica* infected cattle, Sachdev et al. [20] showed that stimulation with LFH induced a significant IL-10 production but a lack of IFN-γ secretion. While in rodent models, an increase of IL-4, IL-10 and IFN-γ production was observed by spleen mononuclear cells during early infection, suggesting a mixed Th1/Th2 response [51].

Antigenic-derived proliferation of CD4^+^ T cells with rFhCB3 and rFhCL2 failed to produce IL-4 and to significantly enhance IFN-γ production during early and late stage of the infection. However, when rhuIL-2 was added to the culture, a slight increase in the IFN-γ level was detected, which again indicates that IL-2 is a restricting factor in the CD4^+^ T cell response. There appear to be multiple mechanisms governing cytokine response that lay on different factors such as the antigen-specific cell stimulation, the immunocompetent cell subsets involved in the adaptive immune response or the time course of the infection which may cause cell exhaustion [20]. For instance, there is evidence to support that one of the key regulatory mechanisms for cytokine production of CD4^+^ T cells is modulated by M2 macrophage-like phenotype induced by *F. hepatica* molecules [22].

As recently reported in cattle [20], we did not detect cell anergy in sheep CD4^+^ T cells in terms of cell proliferation or cytokine production, as demonstrated by the lack of significant differences between naive and infected animals during antigenic stimulation, suggesting a host-dependent mechanism. However, different antigenic stimulation was performed in both studies, which could also explain these contrasting findings.

In conclusion, HLN cells from infected sheep elicited higher significant antigen-specific response to an array of *F. hepatica* derived molecules than uninfected animals which was not detected on CD4^+^ T cells when rFhCB2 and rFhCB3 were used. As hypothesised, no T cell exhaustion was observed on CD4^+^ T cells at the late stage of the infection. This study addressed antigen-specific response to major *F. hepatica*-derived antigens and provides better comprehension of host-parasite interaction.

## Supplementary Information

**Additional file 1: Protein expression profile of *F. hepatica *at different developing stages.**

**Additional file 2: Experiment schedule.**

**Additional file 3: Parasite burden in G5.**

## Data Availability

The datasets during and/or analysed during the current study are available from the corresponding author on reasonable request.

## Abbreviations

APC: Antigen-presenting cells
APCA: Antigen presentation cell assay
HLN: Hepatic lymph node
rFhCL: Recombinant cathepsin L peptidase
rFhCB: Recombinant cathepsin B peptidase
rFhStf: Recombinant stefin cysteine peptidase inhibitor
rFhKT: Recombinant Kunitz type inhibitor
rhuIL-2: Recombinant human interleukin 2
TMB: Tetramethylbenzidine
PBMC: Peripheral blood mononuclear cells
dpi: Days post-infection
wpi: Weeks post-infection
