# Supplementary material for: Antigen-specific response of CD4+ T cells and hepatic lymph node cells to Fasciola hepatica-derived molecules at the early and late stage of the infection in sheep
Source: Vet Res. 2021 Jul 2;52:99. doi: 10.1186/s13567-021-00963-5 (PMC8254349; doi:10.1186/s13567-021-00963-5)
Supplement: Supplementary file 1 — Additional file 1: Protein expression profile of F. hepatica at different developing stages. [file 13567_2021_963_MOESM1_ESM.docx]

| *F. hepatica* molecules | Metacercariae | Newly excysted juvenile fluke | Immature fluke | Adult fluke |
| --- | --- | --- | --- | --- |
| CB1 [25, 26, 27] | ✓ | ✓ | ✓ | - |
| CB2 [25, 26, 27] | ✓ | ✓ | ✓ | - |
| CB3 [25, 26, 27] | ✓ | ✓ | ✓ | - |
| CL1 [25, 27, 28] | - | - | ✓ | ✓ |
| CL2 [25, 27, 28] | - | - | ✓ | ✓ |
| CL3 [25, 27, 28] | ✓ | ✓ | ✓ | - |
| KT1 [25, 26, 29, 30] | ✓ | ✓ | ✓ | ✓ |
| Stf-1[25, 26, 27] | ✓ | ✓ | ✓ | ✓ |
| Stf-2[25, 26, 27] | ✓ | ✓ | ✓ | ✓ |
| Stf-3[25, 26, 27] | ✓ | ✓ | ✓ | ✓ |

**Additional file 1. Protein expression profile of *F. hepatica* at different developing stages**

Expression of *F. hepatica* molecules is indicated (✓), Stf: stefin cysteine peptidase inhibitor. KT: Kunitz type inhibitor. CL: cathepsin L. CB: cathepsin B.
