# Supplementary material for: Antigen-specific response of CD4+ T cells and hepatic lymph node cells to Fasciola hepatica-derived molecules at the early and late stage of the infection in sheep
Source: Vet Res. 2021 Jul 2;52:99. doi: 10.1186/s13567-021-00963-5 (PMC8254349; doi:10.1186/s13567-021-00963-5)
Supplement: Supplementary file 2 — Additional file 2: Experiment schedule. [file 13567_2021_963_MOESM2_ESM.docx]

**Additional file 2 Experiment schedule**

| Activity – Timeline | Trial 1 | | Trial 2 | | |  |
| --- | --- | --- | --- | --- | --- | --- |
|  | G1 (*n* = 8) | G2 (*n* = 8) | G3 (*n* = 5) | G4 (*n* = 5) | G5 (*n* = 5) | |
| Quarantine period (QP) | ✓ | ✓ | ✓ | ✓ | ✓ | |
| Experimental challenge (EC) | - | ✓ | - | ✓ | ✓ | |
| Lymphocyte stimulation assay (LSA) | ✓ | ✓ | - | - | - | |
| Antigen presentation cell assay (APCA) | - | - | ✓ | ✓ | ✓ | |
| Slaughtering at 16 dpi | ✓ | ✓ | - | ✓ | - | |
| Slaughtering at 23 wpi | - | - | ✓ | - | ✓ | |

QR: before commencing the trial, animals were kept for a quarantine period of 30 days. During this period, sheep were treated with ivermectin (Noromectin®, Karizoo, Barcelona, Spain) and diclazuril (Rumicox®, Esteve, Barcelona, Spain) and tested for potential parasite infection by faecal examination before and after treatment, resulting in negative findings. EC: a single infection dose consisting of 150 metacercariae were orally administered to each animal at the beginning of each trial. LSA: a cell proliferation assay using lymphocytes obtained from hepatic lymph nodes and an array of F. hepatica molecules (rFhCL1, rFhCL2, rFhCL3, rFhCB1, rFhCB2, rFhCB3, rFhStf-1, rFhStf-2, rFhStf-3, rFhKT1) was performed. APCA: an antigen presentation cell assay using CD4^+^ T cells (responder cells) and irradiated autologous PBMC (stimulator cells) incubated with *F. hepatica* molecules (rFhCB3 and rFhCL2) was performed. Animals were slaughtered at different time points.
