# Supplementary material for: Antigen-specific response of CD4+ T cells and hepatic lymph node cells to Fasciola hepatica-derived molecules at the early and late stage of the infection in sheep
Source: Vet Res. 2021 Jul 2;52:99. doi: 10.1186/s13567-021-00963-5 (PMC8254349; doi:10.1186/s13567-021-00963-5)
Supplement: Supplementary file 3 — Additional file 3: Parasite burden in G5. [file 13567_2021_963_MOESM3_ESM.docx]

**Additional file 3 Parasite burden in G5**

| Animal | Fluke burden (FB) | Implantation rate (IR, %) |
| --- | --- | --- |
| 1 | 27 | 18.00 |
| 2 | 21 | 14.00 |
| 3 | 28 | 18.67 |
| 4 | 14 | 9.33 |
| 5 | 24 | 16.00 |

FB: numbers of *F. hepatica* adult worms recovered during necropsy, IR: the number of parasites collected from the infection dose (150 metacercariae) is expressed as percentage.
